# Supplementary material for: Health related quality of life and satisfaction with care of stroke patients in Budapest: A substudy of the EuroHOPE project
Source: PLoS One. 2020 Oct 22;15(10):e0241059. doi: 10.1371/journal.pone.0241059 (PMC7580926; doi:10.1371/journal.pone.0241059)
Supplement: S1 Table — (DOCX) [file pone.0241059.s001.docx]

*S1 Table. Predictors of EQ-5D utility index in multiple linear regression model excluding post-stroke variables*

| **EQ-5D utility index (n=117, adjusted R^2^=0.38)** | | | | | |
| --- | --- | --- | --- | --- | --- |
| **Variables** | **Reference** | **Coefficient** | **Lower 95% C.I.** | **Upper 95% C.I.** | **p-value** |
| **Intercept** | - | 1.131 | 0.805 | 1.456 | <0.001 |
| **Sex male** | Female | 0.054 | -0.035 | 0.143 | 0.238 |
| **Age at stroke** | - | -0.010 | -0.014 | -0.006 | <0.001 |
| **Education** | - | 0.007 | -0.009 | 0.023 | 0.405 |
| **NIHSS at admission** | - | -0.022 | -0.033 | -0.012 | <0.001 |
| **TOAST2** | TOAST 1 | 0.239 | 0.098 | 0.380 | 0.001 |
| **TOAST3** |  | 0.206 | 0.074 | 0.338 | 0.003 |
| **TOAST4** |  | 0.359 | 0.000 | 0.717 | 0.053 |
| **TOAST5** |  | 0.140 | 0.003 | 0.276 | 0.048 |
| **Employment prior to stroke: not employed** | Employed | 0.164 | 0.025 | 0.303 | 0.023 |
| **Admitted from other hospital** | Admitted from home | -0.165 | -0.403 | 0.074 | 0.180 |
| **Admitted from other institution** |  | 0.021 | -0.168 | 0.209 | 0.831 |

EQ-5D: the EuroQOL-5 Dimensions-5 Levels questionnaire developed by the EuroQoL group, assessing the health-related quality of life; NIHSS: National Institutes of Health Stroke Scale; TOAST: Trial of Org 10172 in Acute Stroke Treatment.
