# Supplementary material for: Health related quality of life and satisfaction with care of stroke patients in Budapest: A substudy of the EuroHOPE project
Source: PLoS One. 2020 Oct 22;15(10):e0241059. doi: 10.1371/journal.pone.0241059 (PMC7580926; doi:10.1371/journal.pone.0241059)
Supplement: S2 Table — (DOCX) [file pone.0241059.s002.docx]

*S2 Table. Predictors of 15D utility index in multiple linear regression model excluding post-stroke variables*

| **15D** **utility index (n=115, adjusted R^2^=** **0.321)** | | | | | |
| --- | --- | --- | --- | --- | --- |
| **Variables** |  | **Coefficient** | **Lower 95% C.I.** | **Upper 95% C.I.** | **p-value** |
| **Intercept** | - | 1.036 | 0.844 | 1.229 | <0.001 |
| **Sex (male)** | Female | 0.036 | -0.017 | 0.089 | 0.180 |
| **Age at stroke** | - | -0.005 | -0.008 | -0.003 | <0.001 |
| **Education** | - | 0.004 | -0.005 | 0.014 | 0.368 |
| **Admission NIHSS** | - | -0.011 | -0.017 | -0.005 | <0.001 |
| **TOAST2** | TOAST1 | 0.086 | 0.003 | 0.169 | 0.044 |
| **TOAST3** |  | 0.078 | -0.001 | 0.157 | 0.055 |
| **TOAST4** |  | 0.166 | -0.044 | 0.377 | 0.125 |
| **TOAST5** |  | 0.008 | -0.073 | 0.088 | 0.855 |
| **Employment prior to stroke: not employed** | Employed | 0.063 | -0.020 | 0.145 | 0.138 |
| **Admitted from other hospital** | Admitted from home | -0.157 | -0.297 | -0.017 | 0.030 |
| **Admitted from other institution** |  | 0.030 | -0.080 | 0.141 | 0.595 |

15D: the 15-dimension questionnaire assessing the health-related quality of life [26]; NIHSS: National Institutes of Health Stroke Scale; TOAST: Trial of Org 10172 in Acute Stroke Treatment.
