# Supplementary material for: Health related quality of life and satisfaction with care of stroke patients in Budapest: A substudy of the EuroHOPE project
Source: PLoS One. 2020 Oct 22;15(10):e0241059. doi: 10.1371/journal.pone.0241059 (PMC7580926; doi:10.1371/journal.pone.0241059)
Supplement: S3 Table — (DOCX) [file pone.0241059.s003.docx]

*S3 Table. Predictors of PATSAT score and its subcategories in multiple linear regression and ordered logistic regression models excluding post-stroke variables*

| **PATSAT score (n=114, adjusted R^2^=0.028)** | | | | | |
| --- | --- | --- | --- | --- | --- |
| **Variables** | **Reference categories** | **Coefficient** | **Lower 95% C.I.** | **Upper 95% C.I.** | **p-value** |
| **Intercept** | - | 96.709 | 65.916 | 127.502 | <0.001 |
| **Sex male** | Female | 1.941 | -6.336 | 10.219 | 0.647 |
| **Age at stroke** | - | -0.250 | -0.582 | 0.082 | 0.143 |
| **Education** | - | -0.196 | -1.684 | 1.293 | 0.797 |
| **NIHSS at admission** | - | -1.044 | -1.957 | -0.131 | 0.027 |
| **Marital status: living alone** | Living with partner | 1.872 | -6.862 | 10.606 | 0.675 |
| **PATSAT physicians (n=120, adjusted R^2^=0.016)** | | | | | |
| **Variables** | **Reference categories** | **Coefficient** | **Lower 95% C.I.** | **Upper 95% C.I.** | **p-value** |
| **Intercept** | - | 94.162 | 60.977 | 127.347 | <0.001 |
| **Sex male** | Female | -1.063 | -9.967 | 7.841 | 0.815 |
| **Age at stroke** | - | -0.280 | -0.641 | 0.080 | 0.130 |
| **Education** | - | 0.464 | -1.110 | 2.037 | 0.565 |
| **NIHSS at admission** | - | -0.810 | -1.793 | 0.173 | 0.109 |
| **Marital status: living alone** | Living with partner | 2.859 | -6.488 | 12.206 | 0.550 |
| **PATSAT nurses (n=120, adjusted R^2^=0.012)** | | | | | |
| **Variables** | **Reference categories** | **Coefficient** | **Lower 95% C.I.** | **Upper 95% C.I.** | **p-value** |
| **Intercept** | - | 93.122 | 57.509 | 128.735 | <0.001 |
| **Sex male** | Female | 4.732 | -4.889 | 14.353 | 0.337 |
| **Age at stroke** | - | -0.367 | -0.757 | 0.023 | 0.067 |
| **Education** | - | 0.117 | -1.545 | 1.779 | 0.891 |
| **NIHSS at admission** | - | -0.631 | -1.689 | 0.428 | 0.246 |
| **Marital status: living alone** | Living with partner | 0.093 | -10.003 | 10.189 | 0.986 |
| **PATSAT services and care organization (n=116, adjusted R^2^=0.074)** | | | | | |
| **Variables** | **Reference categories** | **Coefficient** | **Lower 95% C.I.** | **Upper 95% C.I.** | **p-value** |
| **Intercept** | - | 82.288 | 49.486 | 115.091 | <0.001 |
| **Sex male** | Female | 3.679 | -4.845 | 12.202 | 0.399 |
| **Age at stroke** | - | -0.095 | -0.441 | 0.250 | 0.589 |
| **Education** | - | 0.614 | -0.883 | 2.110 | 0.423 |
| **NIHSS at admission** | - | -0.953 | -1.893 | -0.014 | 0.049 |
| **Marital status: living alone** | Living with partner | 1.537 | -9.821 | 12.896 | 0.791 |
| **Dwelling prior to stroke: Home alone with help** | Home alone without help | -22.089 | -37.880 | -6.298 | 0.007 |
| **Home with company** |  | -9.157 | -22.111 | 3.797 | 0.169 |
| **In an institution** |  | -18.594 | -46.176 | 8.988 | 0.189 |
| **PATSAT item 32 general satisfaction, ordered logistic regression (n=116, Nagelkerke R^2^=0.104)** | | | | | |
| **Variables** | **Reference categories** | **Odds-ratio** | **Lower 95% C.I.** | **Upper 95% C.I.** | **p-value** |
| **Sex: male** | Female | 1.559 | 0.773 | 3.141 | 0.215 |
| **Age at stroke** |  | 0.978 | 0.949 | 1.008 | 0.146 |
| **Education** |  | 1.027 | 0.907 | 1.164 | 0.671 |
| **NIHSS admission** |  | 0.913 | 0.846 | 0.985 | 0.018 |
| **Marital status: living alone** | Living with partner | 0.914 | 0.443 | 1.886 | 0.808 |

PATSAT: the questionnaire developed by the European Organization for Research and Treatment of Cancer, named EORTC IN-PATSAT32, assessing patient satisfaction; NIHSS: National Institutes of Health Stroke Scale.
