# Supplementary material for: Health related quality of life and satisfaction with care of stroke patients in Budapest: A substudy of the EuroHOPE project
Source: PLoS One. 2020 Oct 22;15(10):e0241059. doi: 10.1371/journal.pone.0241059 (PMC7580926; doi:10.1371/journal.pone.0241059)
Supplement: S4 Table — (DOCX) [file pone.0241059.s004.docx]

*S4 Table.* *Correlation between EQ-5D and 15D utility indices, PATSAT score and its subcategories*

| **First variable** | **Second variable** | **Pearson correlation** | **Pearson p-value** | **Kendall correlation** | **Kendall p-value** | **Spearman correlation** | **Spearman p-value** |
| --- | --- | --- | --- | --- | --- | --- | --- |
| **EQ-5D** | **15D** | 0.792 | <0.001 | 0.650 | <0.001 | 0.825 | <0.001 |
| **EQ-5D** | **PATSAT physicians** | 0.232 | 0.010 | 0.180 | 0.047 | 0.254 | 0.005 |
| **EQ-5D** | **PATSAT nurses** | 0.201 | 0.025 | 0.134 | 0.138 | 0.185 | 0.040 |
| **EQ-5D** | **PATSAT organization** | 0.249 | 0.006 | 0.150 | 0.100 | 0.215 | 0.018 |
| **EQ-5D** | **PATSAT score** | 0.262 | 0.004 | 0.165 | 0.073 | 0.241 | 0.008 |
| **15D** | **PATSAT physicians** | 0.270 | 0.003 | 0.254 | 0.005 | 0.348 | <0.001 |
| **15D** | **PATSAT nurses** | 0.230 | 0.010 | 0.170 | 0.060 | 0.242 | 0.007 |
| **15D** | **PATSAT organization** | 0.248 | 0.006 | 0.177 | 0.053 | 0.249 | 0.006 |
| **15D** | **PATSAT score** | 0.268 | 0.003 | 0.194 | 0.035 | 0.276 | 0.003 |

EQ-5D: the EuroQOL-5 Dimensions-5 Levels questionnaire developed by the EuroQoL group, assessing the health-related quality of life; 15D: the 15-dimension questionnaire assessing health-related quality of life [26]; PATSAT: the questionnaire developed by the European Organization for Research and Treatment of Cancer, named EORTC IN-PATSAT32, assessing patient satisfaction.
