# Supplementary material for: Health related quality of life and satisfaction with care of stroke patients in Budapest: A substudy of the EuroHOPE project
Source: PLoS One. 2020 Oct 22;15(10):e0241059. doi: 10.1371/journal.pone.0241059 (PMC7580926; doi:10.1371/journal.pone.0241059)
Supplement: S5 Table — (DOCX) [file pone.0241059.s005.docx]

*S5 Table. Association between the sub-item of general patient satisfaction (item 32 of the PATSAT questionnaire) and quality of life indices*

| **Global satisfaction** | **EQ-5D utility index mean** | **EQ-5D utility index SD** | **15D utility index mean** | **15D utility index SD** |
| --- | --- | --- | --- | --- |
| **Poor** | 0.544 | 0.267 | 0.655 | 0.262 |
| **Fair** | 0.395 | 0.261 | 0.600 | 0.101 |
| **Good** | 0.646 | 0.325 | 0.735 | 0.155 |
| **Very good** | 0.756 | 0.319 | 0.791 | 0.167 |
| **Excellent** | 0.785 | 0.213 | 0.805 | 0.166 |
| **Kruskal-Wallis test** | chi-squared = 11.763, df = 4,  p-value = 0.019 | | chi-squared = 10.003, df = 4,  p-value = 0.040 | |

EQ-5D: the EuroQOL-5 Dimensions-5 Levels questionnaire developed by the EuroQoL group, assessing the health-related quality of life; 15D: the 15-dimension questionnaire assessing health-related quality of life [26]; PATSAT: the questionnaire developed by the European Organization for Research and Treatment of Cancer, named EORTC IN-PATSAT32, assessing patient satisfaction.
